# Dynamic Divide Grouping Non-Orthogonal Multiple Access in Terrestrial-Satellite Integrated Network

**DOI:** 10.3390/s21186199

**Published:** 2021-09-16

**Authors:** Yanjun Yan, Huihui Xu, Ning Zhang, Guangjie Han, Mingliu Liu

**Affiliations:** 1 The 91892 Troops, Sanya 572000, China; yanj_yan@163.com; 2 School of Weaponry Engineering, Naval University of Engineering, Wuhan 430072, China; 18602710800@163.com; 3 Department of Information and Communication Systems, Hohai University, Changzhou 213000, China; hanguangjie@gmail.com; 4 Electronic Information School, Wuhan University, Wuhan 430072, China; liumingliu@whu.edu.cn

**Keywords:** terrestrial-satellite integrated networks, non orthogonal multiple access, satellite network, stochastic geometry, uplink

## Abstract

Non-orthogonal multiple access (NOMA) has been extensively studied to improve the performance of the Terrestrial-Satellite Integrated Network (TSIN) on account of the shortage of frequency band resources. In this paper, the terrestrial network and satellite network synergistically provide complete coverage for ground users, and based on the architecture, we first formulate a constrained optimization problem to maximize the sum rate of the TSIN under the limited spectrum resources. As the terrestrial networks and the satellite network will cause interference to each other, we first investigate the capacity performance of the terrestrial networks and the satellite networks separately, in which the optimal power control factor expression is derived. Then, by constructing the relationship model between user elevation angle, beam angle and distance, we develop a dynamic group pairing schemes to ensure the effective pairing of NOMA users. Based on the user pairing, to obtain the optimal resource allocation, a joint optimization algorithm of power allocation, beam channel and base station channel resource is proposed. Finally, simulation results are provided to evaluate the user paring scheme as well as the total system performance, in comparison with the existing works.

## 1. Introduction

In the past few decades, the rapid development of terrestrial wireless communications has triggered increasing demand for high broadband and massive access applications, such as augmented reality, virtual reality and high definition video, which in turn raised emergent requirements on achieving massive connectivity and high capacity in future communication systems [1,2]. However, limited by the constrained power storage or insufficient base station deployment, the network coverage in rural or harsh areas is quite low. Due to the wide coverage, broad operating spectrum, and ultra-dense topology, Low Earth Orbit (LEO) satellite networks have been identified as the most cost-affordable technology to meet the the terrestrial coverage requirements [3]. Recently, Terrestrial-Satellite Integrated Network (TSIN) is developed as a combination of satellite networks and terrestrial networks, which have the advantage of providing ubiquitous network services with full frequency reuse to network users [4,5,6].

However, with the exponential growth of wireless connection equipment, the fixed multiple access techniques such as time division multiple access (TDMA), code division multiple access (CDMA) and frequency division multiple access (FDMA), which guarantee orthogonality in time, code and frequency, respectively, will no longer be able to sustain this growing demand for wireless access. To address this challenge in the communication systems, several new techniques for multiple access have recently emerged based on the concept of non-orthogonal multiple access (NOMA) as discussed in [7,8], which can achieve better spectrum utilization and connection density than conventional Orthogonal Multiple Access (OMA) under limited resources. In power-domain NOMA, multiple users can share each subcarrier and the diversity on that subcarrier is obtained by allocating different power levels to the users. The basic principle of NOMA is to exploit the difference in channel gains among users in order to offer multiplexing gains, which can improve the spectral efficiency and massive connectivity.

Despite the proven benefits of NOMA in TSIN [9,10], several practical challenges must be addressed before NOMA can be effectively deployed. First, the base station (BS) and satellite system share the entire bandwidth resources can lead to excessive in-band interference, which will reduce the communication quality of user access. Therefore, to reduce inter-cell interference, it is necessary to consider how to achieve reasonable band selection and power allocation for ground users and satellite users. Second, due to the high dynamic characteristics of satellite, the terrestrial-satellite communication link is time-varying. The user pairing method based on the channel fading model [11] or distance attenuation model [12] will not guarantee a stable pairing relationship. Similarly, by using the antenna gain and quality of service to pair users, the channel difference of users cannot be guaranteed, which restricts the flexibility of satellite user pairing. Therefore, to improve the system throughput, it is necessary to design a NOMA user pairing method for TSIN according to the channel characteristics of terrestrial–satellite link.

Motivated by the advantages of the TSINs, in this paper, aiming at satisfying the high requirements of information capacity, we propose a dynamic divide grouping NOMA method in terrestrial–satellite cooperation system. We consider that, the satellite and the BSs could serve the ground users under coverage cooperatively based on the requirements of them. Moreover, to improve the users throughput, a NOMA problem is modeled as an optimization problem of joint user grouping, power control and resource allocation by using stochastic geometry theory. Different from the aforementioned proposals, we focus on the dynamic characteristic of satellite in actual scenario, by constructing the relationship model between user elevation angle, beam angle and distance, a dynamic divide grouping algorithm is proposed to ensure the effective pairing of NOMA users. Meanwhile, during the solution, to ensure the throughput of cell edge users, based on the instantaneous channel gain, the optimal power control factor expression is derived, then the overall optimal resource allocation is solved by changing the variable form and successive approximation method. The main contributions of this paper are summarized as below:

- To improve user access throughput, we propose a dynamic divide grouping NOMA method for TSINs. Based on the stochastic geometry theory, with the objective of maximizing the network capacity, a NOMA problem is modeled as an optimization problem of joint user grouping, power control and resource allocation.
- To ensure the effective pairing of NOMA users, by constructing the relationship model between user elevation angle, beam angle and distance, a dynamic user pairing algorithm is proposed to pair users into groups for the implementation of NOMA.
- To solve efficiently the problem, based on the instantaneous channel gain, the optimal power control factor expression is derived, then a joint optimization algorithm of beam channel and base station channel resource allocation is proposed.

The remainder of this paper is organized as follows. Section 2 gives the review of related works, then the system architecture and problem formulation are proposed in Section 3. In Section 4, the dynamic divide grouping method is in Section 5. In Section 6, we solve the jointly resource allocation problem of the whole system. The simulation results are shown in Section 6. Finally, Section 7 gives the conclusion.

## 2. Related Work

In the terrestrial networks, the authors of [13,14] studied the influence of user pairing of NOMA system on system performance, and indicated that the larger the difference between the channel conditions for paired users, the higher the capacity of the NOMA system, which verified the influence of NOMA users’ selection strategy on system performance. Combining NOMA technology with a millimeter wave (mmWave) communication model, the authors of [15,16] proposed a user pairing approach based on the mean clustering aiming at maximizing the channel difference. For the sake of the system sum rate, the maximization of the sum rate of a 2-user mmWave NOMA system was investigated in [17], where the authors proposed a NOMA method based on the spatial distribution of users and analyzed the influence of user location on outage probability and throughput. To reduce the computational complexity of user pairing, in [18], the authors proposed a greedy user grouping algorithm to pair users with the largest difference in current channel conditions. However, as the algorithm is local optimal, it can hardly guarantee the channel difference of remaining users. To maximize the difference in channel gains of paired users, a non-orthogonal multiple access algorithm based on dynamic clusters is proposed [19], in which the sum-throughput maximization problem is formulated to optimize user clustering and power allocations in NOMA clusters. The work in [20] presented the throughput performance of NOMA vehicle-to-everything (V2X) with the fixed power allocation factors to further evaluate the performance of a group containing two vehicles. To maximize the sum-capacity and total energy efficiency for small-cell network, the authors of [21] proposed a joint NOMA-enabled optimization framework by utilizing the concepts of the multi-objective problem. Some relatively recent studies also investigate the inventive mechanism in cooperative NOMA and cognitive radio (CR) networks [22,23]. To further improve the performance, a partial relay selection (PRS) technique is adopted to improve the diversity gains and to reduce the overhead of CR-NOMA networks [24].

In the satellite networks, the authors of [25] performed a detailed analysis of coexistence scenarios for 5G and fixed satellite, it showed that 5G can satisfy interference protection criteria of the FSS while allowing simultaneous transmissions from at least several thousands of sectors and tens of thousands of UEs under various LoS and NLoS channel conditions and with various sets of parameters for the fixed satellite service. However, in TSIN, the dynamic of LEO satellite need to be considered for high-speed LEO satellite, a coded random access is employed to realize a high-capacity interface for 5G users, while the slotted Aloha protocol is adopted at the space uplink [26]. However, at the space uplink, the use of slotted Aloha protocol means that it is inevitable that one of the scarce bandwidth resources is solely occupied by this user, despite its poor channel conditions. In such a situation, the use of NOMA ensures not only that the user with poor channel conditions is served, but also that users with better channel conditions can concurrently utilize the same bandwidth resources as the weak user. More recently, the application of NOMA in satellite system have also been investigated. For example, according to the influence of rain attenuation on the link fading, the authors of [27] proposed a heuristic algorithm to solve the optimal power allocation problem of UAV access to satellite. Furthermore, to improve the energy efficiency of satellite, a beamforming algorithm based on iterative penalty function is proposed to maximize the sum capacity of NOMA users [28]. To ensure the channel conditions difference for paired users, a NOMA algorithm based on channel correlation coefficient is proposed [29], where the alternate direction method is used to obtain the suboptimal power allocation. In [30], the author proposed a NOMA method based on millimeter wave, in which the maximum minimum resource allocation problem is modeled as a mixed integer nonlinear programming problem. Further, combining NOMA technology with multiple input multiple output (MIMO) technology, Ding et al. proposed a power allocation approach aiming at maximizing the sum capacity under the interference constraint of the satellite user [31]. In [32,33], the analytical results of outage probability and achievable sum-rate are derived for downlink NOMA in a satellite cell scenario. To improve the system throughput, Jiang et al. [34] proposed a satellite user selection scheme based on channel quality, where the user pairing problem is modeled as a maximum and minimum channel correlation problem and an optimization algorithm is designed to maximize the user communication capacity. In [35], a terrestrial–satellite network model is constructed, where a satellite and numerous BSs served all users, collaboratively. In this model, Lagrange dual method was used to study the power allocation algorithms of terrestrial users and satellite users. To reduce the outage probability of satellite users, Yan et al. proposed a terrestrial–satellite cooperative relay transmission method, which ground node is used to amplify and forward signals [36]. Similarly, in [37], a downlink NOMA mechanism based on user cooperation is proposed, where the user with larger channel gain is used to decode the signals with poor channel conditions. The results show that user cooperation-based NOMA method can improve the reliability and fairness of the system. Based on CR technology, a NOMA algorithm for terrestrial–satellite cognitive network is proposed to improve the spectrum efficiency and the total system capacity [38]. However, CR technology will introduce signal interference between networks, which can leads to the reduced of system throughput.

Table 1 summarizes the core ideas and limitations of the existing methods. It can be observed that the integration of satellite network and terrestrial network will increase the interference between users, resulting in the rate reduction or even communication interruption of cell edge users. Therefore, to reduce the interference between cells, it is necessary to realize the reasonable user pairing selection and power allocation of BS users and satellite users. Similarly, to improve the system throughput, the channel characteristics of satellite–terrestrial link need to be analyzed.

## 3. System Modeling

### 3.1. System Model

**Figure 1:**
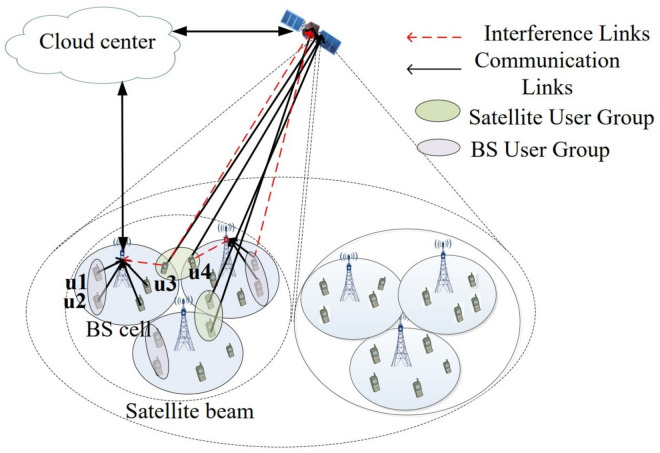
The system model of the non-orthogonal multiple access based integrated terrestrial-satellite network.

The NOMA model of the terrestrial-satellite integrated network is shown in Figure 1, in which BSs and one satellite serve the ground users cooperatively. The low Earth orbit (LEO) satellite is consisted of *L* beam, and each beam is equipped with *M* antennas. Each BS is equipped with *N* antennas for uplink transmission and can serve users within its coverage radius. In this scenario, we consider that the *I* BSs distribute within the coverage of the satellite, and each user can be served either by the corresponding BS or by the satellite. All nodes in the proposed model are also assumed to equip with a single antenna for simplicity. In the TSINs, the NOMA scheme is implemented for multiuser transmission, which can simultaneously serve multiple users with distinct channel conditions reusing the entire bandwidth. As the complexity of multi-user signal detection in NOMA system increases rapidly with the increase of the number of users, and the total system capacity decreases with the increase of the number of users [15], considering both the system load and implementation complexity, the single carrier time-frequency resources are reused for two users to perform NOMA. In the uplink, inter-cell interference comes from all the users in other cells sharing the same subband, as shown in Figure 1. When modeling inter-cell interference, we assume the system is fully loaded and all the cells perform a 2-user uplink NOMA with the same power control scheme. According to the dynamic user pairing scheme, the locations of two users that form a NOMA group in each cell are selected among UEs associated to that cell. For instance, there are *N* users in a single cell and we can select two out of N users in this cell to form a NOMA group on the sub-band under consideration. One of these two users is treated as U1, and the other one is treated as U2. The locations of uses in each cell follows the homogeneous Poisson point process with a intensity $\lambda_{u}$ [39]. In the uplink, a receiver BS or satellite normally has much more capable hardware and advanced algorithms than a UE, so a perfect SIC is assumed at BSs or satellites. Considering that the total power limit can effectively manage inter-group interference, the total power limit of satellite user group *J* is $P_{S,l,J}$, the power control factor of near user and far user in group *J* is $\rho_{S,l,J}^{\left(1\right)}$ and $\rho_{S,l,J}^{\left(2\right)}$, respectively, $\rho_{S,l,J}^{\left(1\right)}+\rho_{S,l,J}^{\left(2\right)}=1$. The bandwidth allocated each group *J* of satellite is $r_{s,l,J}$, and the channel gain between two satellite users (near user and far user) within group *J* is represented as $H_{S,l,J}^{\left(1\right)}$ and $H_{S,l,J}^{\left(2\right)}$, $H_{S,l,J}^{\left(1\right)}>H_{S,l,J}^{\left(2\right)}$, respectively. Similarly, the bandwidth allocated each group *J* in the BS user is defined as $r_{B,i,J}$, $H_{B,i,J}^{\left(1\right)}$ and $H_{B,i,J}^{\left(2\right)}$ represent channel gain between two BS users in group *J*, respectively, $H_{B,i,J}^{\left(1\right)}>H_{B,i,J}^{\left(2\right)}$. $P_{B,i,J}$ denotes the total power limit of BS user group, the power control factor of BS near user and far user is $\rho_{B,i,J}^{\left(1\right)}$ and $\rho_{B,i,J}^{\left(2\right)}$, $\rho_{B,i,J}^{\left(1\right)}+\rho_{B,i,J}^{\left(2\right)}=1$. *n* is the additive white Gaussian noise, which obeys the power spectral density $N_{0}$.

Generally, in current communication networks, BS-based terrestrial communication is more efficient and cheaper than the satellite communication. Thus, in the TSINs, we prefer to maximize the utilization of the BSs, and the satellite will act as the complement to provide service for these users that cannot be served by the BSs. By means of beamforming and NOMA, each BS can provide service for 2N users within its coverage radius, including N near users and N far users. We assume that the total number of users is $2N+k_{i}$, i={1,2...,I}, where 2*N* users are connected to BS, $k_{i}(k_{i}\leq 2M$) users will access satellite in this paper.

The main interference in the integrated network includes inter-system interference and intra-group user interference, among which, the interference of satellite receiving signal mainly comes from the satellite users in the group and BS users with beam coverage, and the receiving signal is expressed as

(1) $$\begin{array}{c} y_{s,l,J}=\left(\sqrt{\rho_{S,l,J}^{\left(1\right)}P_{S},l,JH_{S,l,J}^{\left(1\right)}}X_{S,l,J}^{\left(1\right)}+\sqrt{\rho_{S,l,J}^{\left(2\right)}P_{S},l,JH_{S,l,J}^{\left(2\right)}}X_{S,l,J}^{\left(2\right)}\right)\\ +\sum_{i=1}^{I}\sum_{j=1}^{N}\left(\sqrt{\rho_{B,i,j}^{\left(1\right)}P_{B,i,j}H_{B,i,j}^{\left(1\right)}}X_{B,i,j}^{\left(1\right)}+\sqrt{\rho_{B,i,j}^{\left(2\right)}P_{B,i,j}H_{B,i,j}^{\left(2\right)}}X_{B,i,j}^{\left(2\right)}\right)+n \end{array}$$

The interference of BS receiving signal mainly comes from the BS users in the group and BS users with coverage, and the receiving signal is expressed as

(2) $$\begin{array}{c} y_{B,i,J}=\left(\sqrt{\rho_{B,i,J}^{\left(1\right)}P_{B,i,J}H_{B,i,J}^{\left(1\right)}}X_{B,i,J}^{\left(1\right)}+\sqrt{\rho_{B,i,J}^{\left(2\right)}P_{B,i,J}H_{B,i,J}^{\left(2\right)}}X_{B,i,J}^{\left(2\right)}\right)\\ +\sum_{j=1}^{K_{i}/2}\left(\sqrt{\rho_{S,l,j}^{\left(1\right)}P_{S,l,j}H_{S,l,j}^{\left(1\right)}}X_{S,l,j}^{\left(1\right)}+\sqrt{\rho_{S,l,j}^{\left(2\right)}P_{S,l,j}H_{S,l,j}^{\left(2\right)}}X_{S,l,j}^{\left(2\right)}\right)+n \end{array}$$

### 3.2. Problem Formulation

According to the principle of NOMA, within each group, the NOMA scheme is implemented by applying SIC at the user. The decoding order of SIC is in the order of the increasing channel gain, and thus any user can decode the signals of other users that experience poorer channels than itself. As stated before, the near user experiences a better channel than the far user. The near user will first decode the signal of the far user for interference cancellation, and then decode its own signal after subtracting the far user’s signal from the received signal. Thus, the signal to interference noise ratios (SINR) of two users in the satellite user group *J* (near user and far user) can be calculated as

(3) $$\gamma_{S,l,J}^{\left(1\right)}=\frac{\rho_{S,l,J}^{\left(1\right)}P_{S,l,J}H_{S,l,J}^{\left(1\right)}}{\rho_{S,l,J}^{\left(2\right)}P_{S,l,J}H_{S,l,J}^{\left(2\right)}+I_{B}+r_{S,l,J}N_{0}},$$

(4) $$\gamma_{S,l,J}^{\left(2\right)}=\frac{\rho_{S,l,J}^{\left(2\right)}P_{S,l,J}H_{S,l,J}^{\left(2\right)}}{I_{B}+r_{S,l,J}N_{0}},$$

where $I_{B}=\sum_{i=1}^{I}\sum_{j=1}^{N}\left(\rho_{B,i,j}^{\left(1\right)}P_{B,i,j}H_{B,i,j}^{\left(1\right)}+\rho_{B,i,j}^{\left(2\right)}P_{B,i,j}H_{B,i,j}^{\left(2\right)}\right)$, and the rate expressions of near user and far user in satellite NOMA group can be expressed as

(5) $$C_{S,l,J}^{\left(1\right)}=r_{S,l,J}\log_{2}\left(1+\frac{\rho_{S,l,J}^{\left(1\right)}P_{S,l,J}H_{S,l,J}^{\left(1\right)}}{\rho_{S,l,J}^{\left(2\right)}P_{S,l,J}H_{S,l,J}^{\left(2\right)}+I_{B}+r_{S,l,J}N_{0}}\right),$$

(6) $$C_{S,l,J}^{\left(2\right)}=r_{S,l,J}\log_{2}\left(1+\frac{\rho_{S,l,J}^{\left(2\right)}P_{S,l,J}H_{S,l,J}^{\left(2\right)}}{I_{B}+r_{S,l,J}N_{0}}\right).$$

Furthermore, we can observe that the BS user is interfered by the signals of other satellite users and the BS users. The SINR of the near user and far user in the BS group *J* can be calculated as

(7) $$\gamma_{B,i,J}^{\left(1\right)}=\frac{\rho_{B,i,J}^{\left(1\right)}P_{B,i,J}H_{B,i,J}^{\left(1\right)}}{\rho_{B,i,J}^{\left(2\right)}P_{B,i,J}H_{B,i,J}^{\left(2\right)}+I_{S}+r_{B,i,J}N_{0}},$$

(8) $$\gamma_{B,i,J}^{\left(2\right)}=\frac{\rho_{B,i,J}^{\left(2\right)}P_{B,i,J}H_{B,i,J}^{\left(2\right)}}{I_{S}+r_{B,i,J}N_{0}}.$$

Then, the capacity of the BS near user and BS far user are

(9) $$C_{B,i,J}^{\left(1\right)}=r_{B,i,J}\log_{2}\left(1+\frac{\rho_{B,i,J}^{\left(1\right)}P_{B,i,J}H_{B,i,J}^{\left(1\right)}}{\rho_{B,i,J}^{\left(2\right)}P_{B,i,J}H_{B,i,J}^{\left(2\right)}+I_{S}+r_{B,i,J}N_{0}}\right),$$

(10) $$C_{B,i,J}^{\left(2\right)}=r_{B,i,J}\log_{2}\left(1+\frac{\rho_{B,i,J}^{\left(2\right)}P_{B,i,J}H_{B,i,J}^{\left(2\right)}}{I_{S}+r_{B,i,J}N_{0}}\right),$$

where, $I_{s}=\sum_{j=1}^{K_{i}/2}\left(\rho_{S,l,j}^{\left(1\right)}P_{S,l,j}H_{S,l,j}^{\left(1\right)}+\rho_{S,l,j}^{\left(2\right)}P_{S,l,j}H_{S,l,j}^{\left(2\right)}\right)$.

From the above derivation, we can see that the BSs and the satellite serve multiple ground users cooperatively while NOMA techniques are applied to reuse the entire bandwidth. As the service capability of the BS is limited, the satellite can be utilized to provide extra service for those users that cannot be served by the BSs. However, due to frequency reuse, the terrestrial BSs and the satellite will interfere each other. Furthermore, within each network, there also exists interference among users. Thus, it is of great importance to design the total system reasonably to achieve optimal capacity performance. Considering the communication rate constraints and the transmission power constraints of users, the optimization problem can be formulated as

(11) $$\begin{array}{cc} \max_{r,p} & \sum_{i=1}^{I}\sum_{j=1}^{N}\left(C_{B,i,j}^{\left(1\right)}+C_{B,i,j}^{\left(2\right)}\right)+\sum_{l=1}^{L}\sum_{j=1}^{K_{i}/2}\left(C_{S,l,j}^{\left(1\right)}+C_{S,l,j}^{\left(2\right)}\right),\\ s.t & C1:\sum_{j=1}^{K_{i}/2}r_{S,l,j}\leq r_{S,b},\\ & C2:\sum_{j=1}^{N}r_{B,i,j}\leq r_{B,i,\max},\\ & C3:\rho_{B,i,J}^{\left(1\right)}+\rho_{B,i,J}^{\left(2\right)}=1,\rho_{S,l,J}^{\left(1\right)}+\rho_{S,l,J}^{\left(2\right)}=1,\\ & C4:r_{S,l,j},r_{B,i,j}>0,\\ & C5:\gamma_{S,l,j}^{\left(k\right)},\gamma_{B,i,j}^{\left(k\right)}>\gamma_{\min},k=\{1,2\},\\ & C6:P_{S,l,j},P_{B,i,j}\leq P_{\max}. \end{array}$$

where $r_{S,b}$ is the total resource of satellite, $r_{B,i,\max}$ represents the total satellite resources, $\gamma_{B,i\min}$ refers to the signal to interference noise ratio threshold of cellular user, and $\gamma_{S,l,\min}$ is the signal to interference noise ratio threshold of satellite user. Constraints C1 and C2 denote the total power constraint of the satellite and BS, respectively. Constraint C3 denotes the user power control factor in the group, Constraint C4 ensures the minimum resources of each subchannel, Constraint C5 is the SINR threshold value for satellite users and BS users, and Constraint C6 is the maximum transmit power of the satellite and BS.

As the optimization problem in (11) is non-convex and NP-hard that has NP complexity, which cannot be solved directly. Moreover, as the satellite interferes all the BS users within its coverage, it will lead to a relatively small power allocation for the satellite user if we simply maximize the total capacity. Considering both of the above problems, we decompose the optimization problem into three parts: the dynamic user paring scheme, the terrestrial resource allocation scheme, and the satellite resource allocation scheme. Then, an irrelative algorithm is proposed to optimize the capacity performance of the whole system.

## 4. Dynamic User Pairing Method

Due to the high dynamic characteristics of satellite, the terrestrial–satellite communication link state is time-varying. To ensure the effective pairing of NOMA users, by constructing the relationship model between user elevation angle, beam angle and distance, a dynamic divide grouping algorithm is proposed to pair users into groups for the implementation of NOMA.

For the line-of-sight (LOS) users of communication scenario, as mobile satellite service systems which operate at frequency bands well below 10 GHz in propagation environments suffer from different levels of obstruction [40]. The satellite channel model is composed of beam gain, fading model, and free space loss (FSL) [41], which can be expressed as

(12) $$H_{j}=g_{{}_{j}}\left(\theta \right)G_{j}\left(\phi \right)L_{j},$$

where $L_{j}$ represents the free space loss between satellite and user *j*, j∈{1,2,...,k}. Denoting transmission speed of radio signal as *v*, $h_{j}$ is the distance between the user *j* and satellite, $f_{j}$ denotes the communication frequency, then the FSL $L_{j}$ can be calculated as

(13) $$L_{j}={\left(\frac{v}{4\pi f_{j}h_{j}}\right)}^{2}.$$

Denoting the maximum gain of satellite antenna as $G_{\max}$, $J_{1}\left(.\right)$ is the Bessel function, ϕ denotes the angle between User j and the beam center with respect to the satellite, then the beam gain $G_{j}\left(\phi \right)$ can be expressed as [42]

(14) $$G_{j}\left(\phi \right)=G_{\max}{\left(\frac{J_{1}\left(u_{j}\right)}{2u_{j}}+36\frac{J_{3}\left(u_{j}\right)}{2u_{j}^{3}}\right)}^{2},$$

where $u_{j}=\frac{2.07\sin\phi_{j}}{\sin\phi_{j3dB}}$ [43], $\phi_{j3dB}$ is the 3-dB angle, $\phi =\arctan(\frac{d_{j}}{H_{}})$.

According to the authors of [32], the links between the satellite and terrestrial users undergo independent and identically distributed (i.i.d.) shadowed-Rician fading distribution. The shadowed-Rician fading channel is widely employed in existing literatures because it not only facilitates the mathematic computation but also sufficiently describes the characteristic of satellite terrestrial link. According to the work in [44], the probability density function (PDF) of the Rician fading gain $g_{j_{}}\left(\theta \right)$ is given as

(15) $$f\left(r\right)={\left(\frac{2b_{0}m}{2b_{0}m+\Omega }\right)}^{m}\frac{r}{b_{0}}e^{-\frac{r^{2}}{2b_{0}}}{}_{1}F_{1}\left(m;1;\frac{\Omega r^{2}}{2b_{0}\left(2b_{0}m+\Omega \right)}\right),$$

where ${}_{1}F_{1}\left(a;b;c\right)$ is confluent hypergeometric function, the relations of parameters $b_{0},m,\Omega$ to elevation angles θ is given as

(16) $$\begin{array}{cc} b_{0}\left(\theta \right)= & -4.7963\times 10^{-8}\theta^{3}+5.5784\times 10^{-6}\theta^{2}-2.1344\times 10^{-4}\theta +3.271\times 10^{-2}, \end{array}$$

(17) $$m\left(\theta \right)=6.3739\times 10^{-5}\theta^{3}+5.8533\times 10^{-4}\theta^{2}-1.5973\times 10^{-1}\theta +3.5156,$$

(18) $$\Omega \left(\theta \right)=1.4428\times 10^{-5}\theta^{3}-2.3798\times 10^{-3}\theta^{2}+1.2702\times 10^{-1}\theta -1.4864.$$

**Figure 2:**
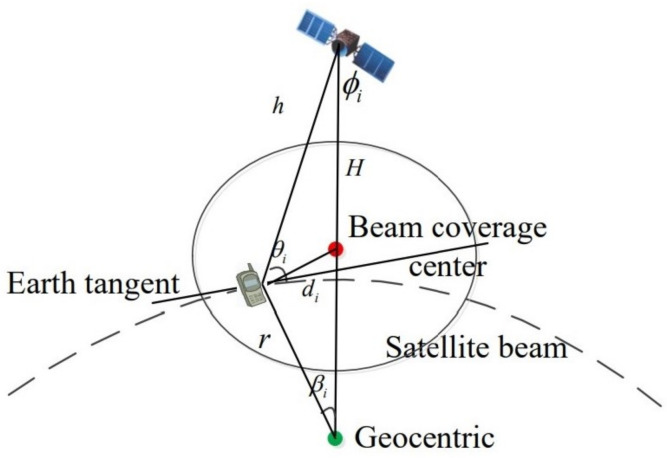
Geometric relationship of beam coverage.

As shown in Figure 2, based on the the side length theorem of triangles, the relationship between $\beta_{i}$ and elevation angles $\theta_{i}$ is expressed as

(19) $$\frac{H+r}{\sin(90^{0}+\theta_{i})}=\frac{h}{\sin\beta_{i}}.$$

Then, the relationship between $\beta_{i}$ and distance $d_{i}$ is given as

(20) $$\frac{\pi r\beta_{i}}{180}=d_{i}.$$

Combining (19) and (20), the relationship between the elevation angles and distance $d_{i}$ can be deduced as follows:

(21) $$\theta_{i}=\arccos\left(\frac{H+r}{h}\sin\left(\frac{180d_{i}}{\pi r}\right)\right).$$

**Figure 3:**
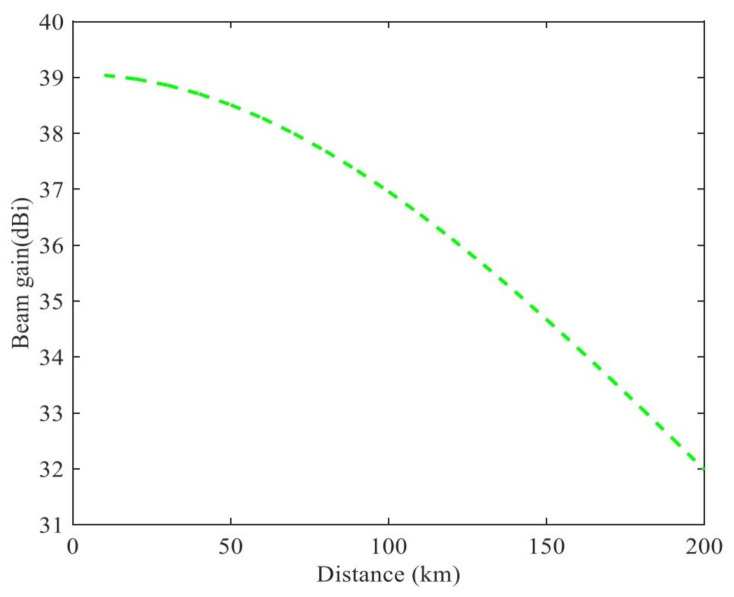
Relationship between beam gain and distance.

Based on the above analysis, the difference of channel gain is mainly determined by the beam gain and the link fading gain. Meanwhile, the larger elevation angle θ, the larger the link fading gain; moreover, the beam gain increases with the decreasing of beam angle Φ. As shown in Figure 3, the beam gain and the $d_{j}$ are presented, $\phi_{j3dB}=14.9^{0}$ [41]. The results show that the difference of satellite communication channel gain is ultimately determined by the distance between the user and the beam center.

**Figure 4:**
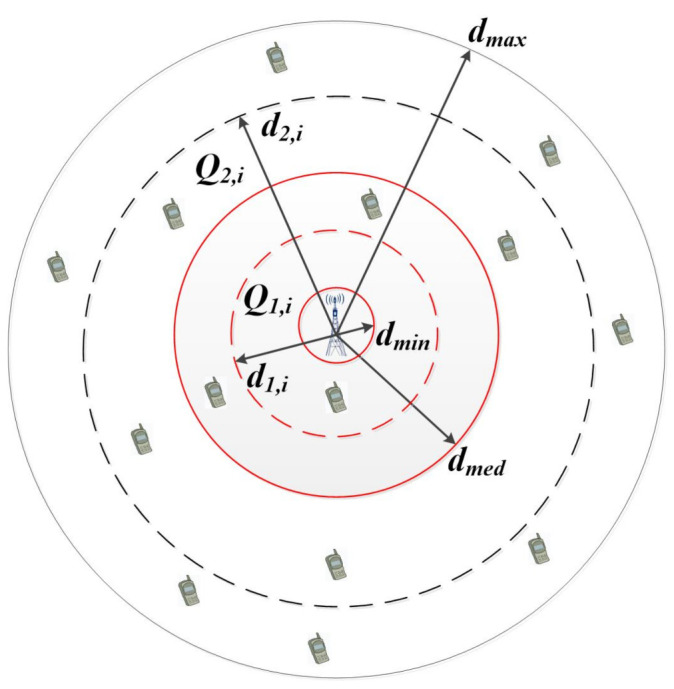
An illustration of dynamic user pairing.

Based on the relationship model of user elevation angle, beam angle and distance, different distance intervals can be divided. Then, the NOMA user pairing with stable channel differences can be constructed by distance model. Specially, the users in each distance interval do not need to sort the channel gain, and the joined users can be pairing according to their distance interval. When the users change dynamically, they only need to determine the changing distance interval and do not need to reorder all user, which can improve the scalability of user pairing. Furthermore, the complexity can be reduced to O(N). As shown in Figure 4, the users are assumed to be randomly deployed in a disc, i.e., the cell controlled by the base station. The radius of the disc is $d_{max}$, and the base station is located at the center. To pair two users for the implementation of NOMA, we assume that the disc is divided into two regions as shown in Figure 4. The first region is a smaller disc with radius $d_{med}$, ($d_{med}\leq d_{max}$) and the base station located at its origin. The second region is a ring, constructed from disc by removing the first region. According to the distance between users and BS or satellite, users are put into different distance intervals to form a set of users. Then, the optimal user pairing is selected according to the interval difference, the specific steps of user pairing are as follows.

(1) Traverse all users $N_{u}$: based on the user’s position, the distance between the user and the BS or the center of the beam coverage area $d_{i}$ is obtained.

(2) Distance interval division: the center point of BS or beam coverage area is shown in Figure 4, the user terminal density is $\lambda_{u}$, which follows the homogeneous Poisson point process. Denoting $d_{med}$ as the distance corresponding to the users number $\frac{N_{u}}{2}$, according to the user distribution, the interval $\left[d_{\min},d_{\max}\right]$ is divided into two intervals, which are expressed as $Q_{1}=\left[d_{\min},d_{med}\right]$ and $Q_{2}=\left[d_{med},d_{\max}\right]$. The users number in $Q_{1}$ is $P\left(N_{k}=k\right)=\frac{\mu_{1}^{k}}{K!}e^{-\mu_{1}}$, $\mu_{1}=\pi \left(d_{med}^{2}-d_{\min}^{2}\right)\lambda_{\mu }$, and the users number in $Q_{2}$ is $P\left(N_{k}=k\right)=\frac{\mu_{2}^{k}}{K!}e^{-\mu_{2}}$, $\mu_{2}=\pi \left(d_{\max}^{2}-d_{med}^{2}\right)\lambda_{\mu }$.

Each interval obtained from the first level partition is divided into two levels, and *q* is defined as the number of partitions. To ensure the equal number of users in each interval, we have

(22) $$\frac{\mu_{i}^{k}}{K!}e^{-\mu_{i}}=\frac{N_{u}}{2q}.$$

with

(23) $$\mu_{i}=\pi \left(d_{1,i}^{2}-d_{1,i-1}^{2}\right)\lambda_{\mu }.$$

According to the number of users $\frac{N_{u}}{2q}$ corresponding to the interval, the distance boundary value of the corresponding interval $\left[d_{i-1},d_{i}\right]$ can be obtained, i.e, the subinterval *i*(1≤i≤q) in the first interval is expressed as $Q_{1,i}=\left[d_{1,i-1},d_{1,i}\right]$, the subinterval *i* in the second interval is expressed as $Q_{2,i}=\left[d_{2,i-1},d_{2,i}\right]$.

(3) Partition user set: traverse all users and divide them into corresponding intervals according to channel gain, then a user set $S_{i,j}$ can be obtained.

(4) Generate user pairing: according to the interval index, the user set of secondary interval is paired. To select the distance group with the largest channel difference and avoid the fairness problem caused by uneven distance distribution, the optimal distance interval pairing need to satisfy that

(24) $$\begin{array}{cc} \max_{} & \sum_{i\in d_{1,i},j\in d_{1,j}}^{}|d_{1,i}-d_{1,j}| \end{array},$$

(25) $$\min\begin{array}{cc} & \sqrt{\sum_{i}^{}{\left|\Delta d_{i,j}-\bar{d_{i,j}}\right|}^{2}} \end{array},$$

where $\Delta d_{i,j}=\left|d_{1,i}-d_{1,j}\right|$ , $\bar{d_{i,j}}=\frac{2\sum_{i\in d_{1,i},j\in d_{1,j}}^{}|d_{1,i}-d_{1,j}|}{N}$.

Then, the composition of a NOMA user pairing can be expressed as

(26) $$G_{j}=\left\{d_{i}\in Q_{1,j},d_{j}\in Q_{2,j},1\leq i\leq q,1\leq j\leq q\right\}.$$

**Figure 5:**
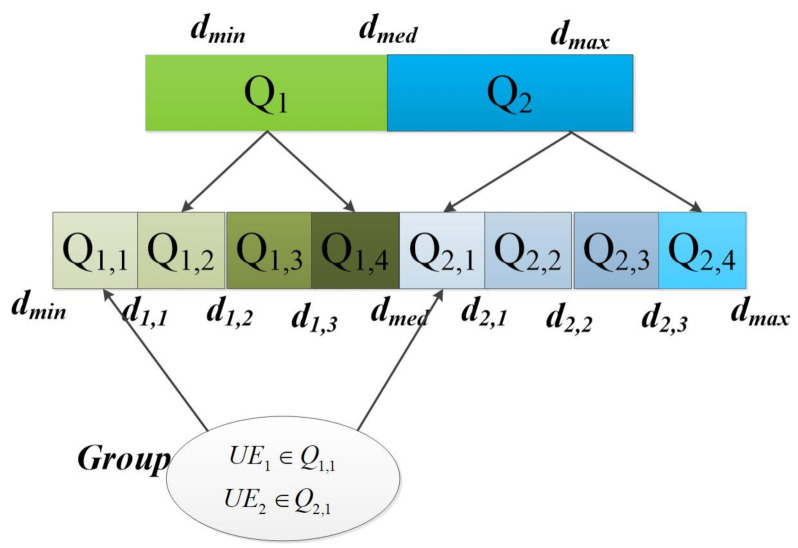
User pairing for uplink NOMA.

As shown in Figure 5, the diagram of user pairing when q = 4 is presented. First, according to the distance between users and BS or satellite, users are put into different distance intervals. Then, the optimal user pairing is selected according to the interval difference in step 4.

## 5. Joint Resource Allocation Optimization Scheme

Due to the application of NOMA and complicated objective function, the original problem in (11) is complex; therefore, we propose a power control factor optimization and power allocation scheme to obtain the optimal solutions.

### 5.1. Power Control Factor Optimization

For many practical scenarios, the total transmission power constraint is a crucial criterion. For example, in a cell with multiple users sharing the same bandwidth, the constraint of the total transmission power within this cell is important to manage inter-cell interference. Another example is hybrid NOMA, where users are paired to perform NOMA and inter-pair interference is canceled by relying on conventional interference management techniques. The use of the total power constraint is therefore useful to measure inter-group interference [45], i.e., $\rho_{S,l,J}^{\left(1\right)}+\rho_{S,l,J}^{\left(2\right)}=1$. To reduce the interference of users in the group and ensure the lowest transmission rate of users, the optimal power control factor is designed to achieve higher resource utilization and improve the total throughput of users.

On the other hand, the rate of user *i* in OMA, such as time division multiple access (TDMA), is given by

(27) $$R_{T}^{\left(i\right)}=\frac{1}{2}\log_{2}\left(1+\frac{P_{B,i,J}H_{B,i,J}^{\left(i\right)}}{I_{S}+r_{B,i,J}N_{0}}\right),i=\left\{1,2\right\}.$$

To assure that the transmission rate with the NOMA scheme always outperforms that with the TDMA scheme, users 1 and 2 in a group need to satisfy that $R_{B,i,J}^{\left(i\right)}\geq R_{T}^{\left(i\right)}$, i.e.,

(28) $$\log_{2}\left(1+\frac{\rho_{B,i,J}^{\left(1\right)}P_{B,i,J}H_{B,i,J}^{\left(1\right)}}{\rho_{B,i,J}^{\left(2\right)}P_{B,i,J}H_{B,i,J}^{\left(2\right)}+I_{S}+r_{B,i,J}N_{0}}\right)\geq \frac{1}{2}\log_{2}\left(1+\frac{P_{B,i,J}H_{B,i,J}^{\left(1\right)}}{P_{B,i,J}H_{B,i,J}^{\left(2\right)}+I_{S}+r_{B,i,J}N_{0}}\right).$$

(29) $$\log_{2}\left(1+\frac{\rho_{B,i,J}^{\left(2\right)}P_{B,i,J}H_{B,i,J}^{\left(2\right)}}{I_{S}+r_{B,i,J}N_{0}}\right)\geq \frac{1}{2}\log_{2}\left(1+\frac{P_{B,i,J}H_{B,i,J}^{\left(2\right)}}{I_{S}+r_{B,i,J}N_{0}}\right).$$

Due to $\rho_{B,i,J}^{\left(1\right)}+\rho_{B,i,J}^{\left(2\right)}=1$, the range of power allocation factor can be further constrained as

(30) $$\frac{1+\frac{P_{B,i,J}H_{B,i,J}^{\left(2\right)}}{I_{S}+r_{B,i,J}N_{0}}}{1+\frac{P_{B,i,J}H_{B,i,J}^{\left(2\right)}}{I_{S}+r_{B,i,J}N_{0}}+\sqrt{1+\frac{P_{B,i,J}H_{B,i,J}^{\left(1\right)}}{I_{S}+r_{B,i,J}N_{0}}}+1}\leq \rho_{B,i,J}^{\left(1\right)}\leq \frac{\sqrt{1+\frac{P_{B,i,J}H_{B,i,J}^{\left(2\right)}}{I_{S}+r_{B,i,J}N_{0}}}}{\sqrt{1+\frac{P_{B,i,J}H_{B,i,J}^{\left(2\right)}}{I_{S}+r_{B,i,J}N_{0}}}+1}.$$

Then, the sum rate of group users can be expressed as

(31) $$R_{sum}\left(\rho_{B,i,J}^{\left(1\right)}\right)=\log_{2}\left(1+\frac{\rho_{B,i,J}^{\left(1\right)}P_{B,i,J}H_{B,i,J}^{\left(1\right)}}{\left(1-\rho_{B,i,J}^{\left(1\right)}\right)P_{B,i,J}H_{B,i,J}^{\left(2\right)}+I_{S}+r_{B,i,J}N_{0}}\right)+\log_{2}\left(1+\frac{\left(1-\rho_{B,i,J}^{\left(1\right)}\right)P_{B,i,J}H_{B,i,J}^{\left(2\right)}}{I_{S}+r_{B,i,J}N_{0}}\right).$$

Let $k_{1}=P_{B,i,J}H_{B,i,J}^{\left(1\right)},k_{2}=P_{B,i,J}H_{B,i,J}^{\left(2\right)},b=I_{S}+r_{B,i,J}N_{0}$, the first derivative of sum rate $R_{sum}\left(\rho_{B,i,J}^{\left(1\right)}\right)$ with respect to $\rho_{B,i,J}^{\left(1\right)}$ is

(32) $$R_{sum}{\left(\rho_{B,i,J}^{\left(1\right)}\right)}^{{}^{\prime }}=\frac{\left(k_{1}k_{2}-{k_{2}}^{2}\right)\left(1-\rho_{B,i,J}^{\left(1\right)}\right)+b\left(k_{1}-k_{2}\right)}{\left[\left(1-\rho_{B,i,J}^{\left(1\right)}\right)k_{2}+b\right]\left(1+\rho_{B,i,J}^{\left(1\right)}k_{1}\right)}.$$

Due to $H_{B,i,J}^{\left(1\right)}>H_{B,i,J}^{\left(2\right)}$, the first derivative of sum rate $R_{sum}\left(\rho_{B,i,J}^{\left(1\right)}\right)$ with respect to $\rho_{B,i,J}^{\left(1\right)}$ is strictly positive, which implies that the sum rate is increasing with $\rho_{B,i,J}^{\left(1\right)}$. Therefore, taking the constraint of (30) into consideration, the maximized sum rate of group users is obtained when $\rho_{B,i,J}^{\left(1\right)}=\frac{\sqrt{1+\frac{P_{B,i,J}H_{B,i,J}^{\left(2\right)}}{I_{S}+r_{B,i,J}N_{0}}}}{\sqrt{1+\frac{P_{B,i,J}H_{B,i,J}^{\left(2\right)}}{I_{S}+r_{B,i,J}N_{0}}}+1}$.

Similarly, the intra-group power allocation of the satellite will satisfy that

(33) $$\log_{2}\left(1+\frac{\rho_{S,l,J}^{\left(1\right)}P_{S,l,J}H_{S,l,J}^{\left(1\right)}}{\rho_{S,l,J}^{\left(2\right)}P_{S,l,J}H_{S,l,J}^{\left(2\right)}+I_{B}+r_{S,l,J}N_{0}}\right)\geq \frac{1}{2}\log_{2}\left(1+\frac{P_{S,l,J}H_{S,l,J}^{\left(1\right)}}{P_{S,l,J}H_{S,l,J}^{\left(2\right)}+I_{B}+r_{S,l,J}N_{0}}\right),$$

(34) $$\log_{2}\left(1+\frac{\rho_{S,l,J}^{\left(2\right)}P_{S,l,J}H_{S,l,J}^{\left(2\right)}}{I_{B}+\sigma^{2}}\right)\geq \frac{1}{2}\log_{2}\left(1+\frac{P_{S,l,J}H_{S,l,J}^{\left(2\right)}}{I_{B}+\sigma^{2}}\right).$$

Due to $\rho_{S,l,J}^{\left(1\right)}+\rho_{S,l,J}^{\left(2\right)}=1$, we have

(35) $$\frac{1+\frac{P_{S,l,J}H_{S,l,J}^{\left(2\right)}}{I_{B}+r_{S,l,J}N_{0}}}{1+\frac{P_{S,l,J}H_{S,l,J}^{\left(2\right)}}{I_{B}+r_{S,l,J}N_{0}}+\sqrt{1+\frac{P_{S,l,J}H_{S,l,J}^{\left(1\right)}}{I_{B}+r_{S,l,J}N_{0}}}+1}\leq \rho_{S,l,J}^{\left(1\right)}\leq \frac{\sqrt{1+\frac{P_{S,l,J}H_{S,l,J}^{\left(2\right)}}{I_{B}+\sigma^{2}}}}{\sqrt{1+\frac{P_{S,l,J}H_{S,l,J}^{\left(2\right)}}{I_{B}+\sigma^{2}}}+1}.$$

The maximized sum rate of satellite group users can be obtained when $\rho_{S,l,J}^{\left(1\right)}=\frac{\sqrt{1+\frac{P_{S,l,J}H_{S,l,J}^{\left(2\right)}}{I_{B}+r_{S,l,J}N_{0}}}}{\sqrt{1+\frac{P_{S,l,J}H_{S,l,J}^{\left(2\right)}}{I_{B}+r_{S,l,J}N_{0}}}+1}$.

### 5.2. Satellite Beam Channel Resource Allocation

After determining the intra-group power allocation based on the above analysis, to maximize the capacity performance of the satellites, it is important to implement reasonable power allocation among groups under the constraint of total power. As frequency reuse is considered in the system, there will be inter-group interference and also need to avoid large interference to BS users, the optimization problem of satellite beam channel resource allocation can be formulated as

(36) $$\begin{array}{c} \max_{r,p}\begin{array}{cc} & \sum_{J=1}^{K_{i}/2}\left(C_{S,l,J}^{\left(1\right)}+C_{S,l,J}^{\left(2\right)}\right) \end{array},\\ \begin{array}{cc} s.t & C1:\sum_{j=1}^{K_{i}/2}r_{S,l,J} \end{array}\leq r_{S,b},\\ \begin{array}{ccc} & & C2:r_{S,l,J},P_{S,l,J}>0, \end{array}\\ \begin{array}{ccc} & & C3:P_{S,l,j}\leq P_{\max}. \end{array} \end{array}$$

where $r_{S,b}$ is the total resource of satellite, $r_{S,l,j}$ represents the allocated resource for the beam’s sub channel *j* and $P_{\max}$ represents the maximum power transmitted of group *J*. Constraint C1 denotes the power constraint of the satellite beam, Constraint C2 ensures the minimum resources of each group and Constraint C3 is the maximum transmit power of the group *j*.

As the optimization problem is nonconvex, according to successive approximation method [46], the objective function can be transformed as

(37) $$r_{S,l,J}\log_{2}\left(1+\gamma_{S,l,J}^{\left(1\right)}\right)\geq \frac{r_{S,l,J}}{In2}\left(\theta_{S,l,J}^{\left(1\right)}In\gamma_{S,l,J}^{\left(1\right)}+\beta_{S,l,J}^{\left(1\right)}\right),$$

(38) $$r_{S,l,J}\log_{2}\left(1+\gamma_{S,l,J}^{\left(2\right)}\right)\geq \frac{r_{S,l,J}}{In2}\left(\theta_{S,l,J}^{\left(2\right)}In\gamma_{S,l,J}^{\left(2\right)}+\beta_{S,l,J}^{\left(2\right)}\right),$$

in which approximation parameters are calculated as

(39) $$\theta_{S,l,J}^{\left(1\right)}=\frac{\bar{\gamma_{S,l,J}^{\left(1\right)}}}{1+\bar{\gamma_{S,l,J}^{\left(1\right)}}},\beta_{S,l,J}^{\left(1\right)}=In\left(1+\bar{\gamma_{S,l,J}^{\left(1\right)}}\right)-\frac{\bar{\gamma_{S,l,J}^{\left(1\right)}}}{1+\bar{\gamma_{S,l,J}^{\left(1\right)}}}In\bar{\gamma_{S,l,J}^{\left(1\right)}},$$

(40) $$\theta_{S,l,J}^{\left(2\right)}=\frac{\bar{\gamma_{S,l,J}^{\left(2\right)}}}{1+\bar{\gamma_{S,l,J}^{\left(2\right)}}},\beta_{S,l,J}^{\left(2\right)}=In\left(1+\bar{\gamma_{S,l,J}^{\left(2\right)}}\right)-\frac{\bar{\gamma_{S,l,J}^{\left(2\right)}}}{1+\bar{\gamma_{S,l,J}^{\left(2\right)}}}In\bar{\gamma_{S,l,J}^{\left(2\right)}}.$$

Then, as $\sum_{J=1}^{K_{i}/2}\frac{r_{S,l,J}}{In2}[\left(\theta_{S,l,J}^{\left(1\right)}In\gamma_{S,l,J}^{\left(1\right)}+\beta_{S,l,J}^{\left(1\right)}\right)+\left(\theta_{S,l,J}^{\left(2\right)}In\gamma_{S,l,J}^{\left(2\right)}+\beta_{S,l,J}^{\left(2\right)}\right)]$ is the lower bound of $\sum_{J=1}^{K_{i}/2}\left(C_{S,l,J}^{\left(1\right)}+C_{S,l,J}^{\left(2\right)}\right)$, changing the variable $\overset{-}{P_{S,l,J}}=InP_{S,l,J}$, the optimization problem in each iteration is transformed into

(41) $$\begin{array}{c} \max\begin{array}{cc} & \sum_{J=1}^{K_{i}/2}\frac{r_{S,l,J}}{In2}\left[\left(\theta_{S,l,J}^{\left(1\right)}In\overset{-}{\gamma_{S,l,J}^{\left(1\right)}}+\beta_{S,l,J}^{\left(1\right)}\right)+\left(\theta_{S,l,J}^{\left(2\right)}In\overset{-}{\gamma_{S,l,J}^{\left(2\right)}}+\beta_{S,l,J}^{\left(2\right)}\right)\right], \end{array}\\ \begin{array}{cc} s.t & C1:\sum_{j=1}^{K_{i}/2}r_{S,l,J} \end{array}\leq r_{S,b},\\ \begin{array}{ccc} & & C2:r_{S,l,J},e^{\overset{-}{P_{S,l,J}}}>0, \end{array}\\ \begin{array}{ccc} & & C3:e^{\overset{-}{P_{S,l,J}}}\leq P_{\max}. \end{array} \end{array}$$

As the log-sum-exp function is convex [46], it is easy to prove the transformed subproblem in each iteration is a standard convex optimization problem. However, by solving the subproblem, we only obtain the lower bound of the capacity. To solve the original problem (36), the maximum the lower bound $\sum_{J=1}^{K_{i}/2}\frac{r_{S,l,J}}{In2}[\left(\theta_{S,l,J}^{\left(1\right)}In\overset{-}{\gamma_{S,l,J}^{\left(1\right)}}+\beta_{S,l,J}^{\left(1\right)}\right)+\left(\theta_{S,l,J}^{\left(2\right)}In\overset{-}{\gamma_{S,l,J}^{\left(2\right)}}+\beta_{S,l,J}^{\left(2\right)}\right)]$ is obtained by dual decomposition algorithm [46]. By using (39) and (40), we iteratively update the approximation parameters $\left(\theta_{S,l,J}^{},\beta_{S,l,J}^{}\right)$, then use the updated parameters for the next iteration until the results converge, as stated in the SCA approach above.

In each iteration, we solve the subproblem (41) by means of the Lagrangian dual method. The Lagrangian function is

(42) $$\begin{array}{c} L\left(r_{S,l,J},\overset{-}{P_{S,l,J}},\lambda ,\mu \right)=\sum_{J=1}^{K_{i}/2}\frac{r_{S,l,J}}{In2}[\left(\theta_{S,l,J}^{\left(1\right)}In\gamma_{S,l,J}^{\left(1\right)}+\beta_{S,l,J}^{\left(1\right)}\right)+\left(\theta_{S,l,J}^{\left(2\right)}In\gamma_{S,l,J}^{\left(2\right)}+\beta_{S,l,J}^{\left(2\right)}\right)]\\ \begin{array}{cccc} & & & +\lambda \left(r_{S,b}-\sum_{j=1}^{K_{i}/2}r_{S,l,J}\right)+\mu \left(P_{\max}-e^{\overset{-}{P_{S,l,J}}}\right) \end{array} \end{array}.$$

where λ,μ the Lagrange multiplier for the constraint condition, the dual problem can be expressed as

(43) $$\begin{array}{c} \begin{array}{cc} \min_{\lambda ,\mu } & \max L\left(r_{S,l,J},\overset{-}{P_{S,l,J}},\lambda ,\mu \right), \end{array}\\ \begin{array}{cc} s.t & C1-C3. \end{array} \end{array}$$

Based on the standard KKT condition [46], by deriving $r_{S,l,J}$ and $\overset{-}{P_{S,l,J}}$, we can obtain the optimal power allocation scheme of the subproblem in the form of λ and μ

(44) $$P_{S,l,J}\left[t+1\right]=e^{\overset{-}{P_{S,l,J}\left[t+1\right]}}={\left[\frac{\theta_{S,l,J}^{\left(1\right)}+\theta_{S,l,J}^{\left(2\right)}}{\mu In2+\frac{\theta_{S,l,J}^{\left(1\right)}\rho_{S,l,J}^{\left(1\right)}H_{S,l,J}^{\left(1\right)}}{\rho_{S,l,J}^{\left(2\right)}H_{S,l,J}^{\left(2\right)}e^{\overset{-}{P_{S,l,J}\left[t\right]}}+I_{B}+r_{S,l,J}\left[t\right]N_{0}}}\right]}^{+},$$

(45) $$r_{S,l,J}\left[t+1\right]={\left[\frac{\lambda In2-\frac{\theta_{S,l,J}^{\left(1\right)}\rho_{S,l,J}^{\left(1\right)}H_{S,l,J}^{\left(1\right)}e^{\overset{-}{P_{S,l,J}}\left[t\right]}}{\rho_{S,l,J}^{\left(2\right)}H_{S,l,J}^{\left(2\right)}e^{\overset{-}{P_{S,l,J}\left[t\right]}}+I_{B}+r_{S,l,J}\left[t\right]N_{0}}-\frac{\theta_{S,l,J}^{\left(2\right)}\rho_{S,l,J}^{\left(2\right)}H_{S,l,J}^{\left(2\right)}e^{\overset{-}{P_{S,l,J}\left[t\right]}}}{I_{B}+r_{S,l,J}\left[t\right]N_{0}}}{N_{0}e^{\overset{-}{P_{S,l,J}\left[t\right]}}\left(\theta_{S,l,J}^{\left(1\right)}\rho_{S,l,J}^{\left(1\right)}H_{S,l,J}^{\left(1\right)}+\theta_{S,l,J}^{\left(2\right)}\rho_{S,l,J}^{\left(2\right)}H_{S,l,J}^{\left(2\right)}\right)}\right]}^{+}.$$

where ${\left[x\right]}^{+}=\max\left(0,x\right)$.

As $L(r_{S,l,J},\overset{-}{P_{S,l,J}},\lambda ,\mu )$ in (42) is not differentiable, the Lagrange multiplier λ and μ can be obtained by using the subgradient method iteratively as follows:

(46) $$\mu \left[t_{\delta }+1\right]={\left[\mu \left[t_{\delta }\right]-\delta_{\mu }\left(P_{\max}-P_{S,l,J}\right)\right]}^{+},$$

(47) $$\lambda \left[t_{\delta }+1\right]={\left[\lambda \left[t_{\delta }\right]-\delta_{\lambda }\left(r_{S,b}-\sum_{j=1}^{K_{i}/2}r_{S,l,J}\right)\right]}^{+},$$

where $\delta_{\lambda }$ and $\delta_{\mu }$ are the step size of λ and μ in each iteration. The satellite beam channel allocation scheme is summarized as Algorithm 1. In the outer loop, we update the approximation parameters $\theta_{S,l,J}^{}$ and $\beta_{S,l,J}^{}$, and transform the original optimization problem into a solvable convex problem by logarithmic approximation. In the inter loop, we solve the transformed subproblem using the Lagrangian dual method. By updating the approximation parameters iteratively, we can finally obtain the optimal solution of original problem in (41).

### 5.3. BS Channel Resource Allocation

To maximize the capacity performance of BS users, by fixing the satellite parameters, we study the channel allocation scheme for the BS users. As stated before, the satellite will cause interference to all the BS users within its coverage. Thus, the optimization problem can be formulated as follows:    

(48) $$\begin{array}{c} \max_{r,p}\begin{array}{cc} & \sum_{j=1}^{N}\left(C_{B,i,j}^{\left(1\right)}+C_{B,i,j}^{\left(2\right)}\right), \end{array}\\ \begin{array}{cc} s.t & C1:\sum_{j=1}^{N}r_{B,i,j}\leq r_{B,i,\max}, \end{array}\\ C2:r_{B,i,j},P_{B,i,j}>0,\\ C3:P_{B,i,j}\leq P_{\max}. \end{array}$$

where $r_{B,i,\max}$ is the total resource of BS, $r_{B,i,j}$ represents the allocated resource for the BS subchannel *j* and $P_{\max}$ represents the maximum power transmitted of group *J*. Constraint C1 denotes the power constraint of the BS, Constraint C2 ensures the minimum resources of each group and Constraint C3 is the maximum transmit power of the group *j*.
**Algorithm 1** Satellite Beam Channel Resource Allocation.1: **Initialize:** t=1,$\theta_{S,l,J}^{\left(1\right)}=\theta_{S,l,J}^{\left(2\right)}=1,\beta_{S,l,J}^{\left(1\right)}=\beta_{S,l,J}^{\left(2\right)}=0$; Initialize power $P_{S,l,J}=0$; 2: **repeat** 3: Initialize $t_{\delta }=1$,λ>0,μ>0 4: Initialize $I_{B}$ 5:    **repeat** 6:       **for** J=1 to $K_{i}/2$ do 7:          Update $P_{S,l,J}$ by using (44) 8:          Update $r_{S,l,J}$ by using (45) 9:       **end for** 10:       Update λ,μ by using (46) and (47) 11:       Set $t_{\delta }=t_{\delta }+1$ 12:       Until λ,μ converges 13: Set $P_{S,l,J}\left[t\right]=P_{S,l,J}\left[t+1\right]$ 14: Update $\theta_{S,l,J}^{\left(1\right)},\theta_{S,l,J}^{\left(2\right)},\beta_{S,l,J}^{\left(1\right)},\beta_{S,l,J}^{\left(2\right)}$ by using (39) and (40) 15: Set t=t+1 16: Until $P_{S,l,J}$, $r_{S,l,J}$ converges 

Similarly, according to successive approximation method [46], the objective function can be transformed as

(49) $$r_{B,i,j}\log_{2}\left(1+\gamma_{B,i,j}^{\left(1\right)}\right)\geq \frac{r_{B,i,j}}{In2}\left(\theta_{B,i,j}^{\left(1\right)}In\gamma_{B,i,j}^{\left(1\right)}+\beta_{B,i,j}^{\left(1\right)}\right),$$

(50) $$r_{B,i,j}\log_{2}\left(1+\gamma_{B,i,j}^{\left(2\right)}\right)\geq \frac{r_{B,i,j}}{In2}\left(\theta_{B,i,j}^{\left(2\right)}In\gamma_{B,i,j}^{\left(2\right)}+\beta_{B,i,j}^{\left(2\right)}\right),$$

where approximation parameters are calculated as

(51) $$\theta_{B,i,j}^{\left(1\right)}=\frac{\bar{\gamma_{B,i,j}^{\left(1\right)}}}{1+\bar{\gamma_{B,i,j}^{\left(1\right)}}},\beta_{B,i,j}^{\left(1\right)}=In\left(1+\bar{\gamma_{B,i,j}^{\left(1\right)}}\right)-\frac{\bar{\gamma_{B,i,j}^{\left(1\right)}}}{1+\bar{\gamma_{B,i,j}^{\left(1\right)}}}In\bar{\gamma_{B,i,j}^{\left(1\right)}},$$

(52) $$\theta_{B,i,j}^{\left(2\right)}=\frac{\bar{\gamma_{B,i,j}^{\left(2\right)}}}{1+\bar{\gamma_{B,i,j}^{\left(2\right)}}},\beta_{B,i,j}^{\left(2\right)}=In\left(1+\bar{\gamma_{B,i,j}^{\left(2\right)}}\right)-\frac{\bar{\gamma_{B,i,j}^{\left(2\right)}}}{1+\bar{\gamma_{B,i,j}^{\left(2\right)}}}In\bar{\gamma_{B,i,j}^{\left(2\right)}}.$$

Then, as $\sum_{j=1}^{N}\frac{r_{B,i,j}}{In2}[\left(\theta_{B,i,j}^{\left(1\right)}In\gamma_{B,i,j}^{\left(1\right)}+\beta_{B,i,j}^{\left(1\right)}\right)+\left(\theta_{B,i,j}^{\left(2\right)}In\gamma_{B,i,j}^{\left(2\right)}+\beta_{B,i,j}^{\left(2\right)}\right)]$ is the lower bound of $\sum_{j=1}^{N}\left(C_{B,i,j}^{\left(1\right)}+C_{B,i,j}^{\left(2\right)}\right)$, changing the variable $\overset{-}{P_{B,i,j}}=InP_{B,i,j}$, the optimization problem in each iteration is transformed into

(53) $$\begin{array}{c} \max\begin{array}{cc} & \sum_{j=1}^{N}\frac{r_{B,i,j}}{In2}\left[\left(\theta_{B,i,j}^{\left(1\right)}In\overset{-}{\gamma_{B,i,j}^{\left(1\right)}}+\beta_{B,i,j}^{\left(1\right)}\right)+\left(\theta_{B,i,j}^{\left(2\right)}In\overset{-}{\gamma_{B,i,j}^{\left(2\right)}}+\beta_{B,i,j}^{\left(2\right)}\right)\right] \end{array}\\ \begin{array}{cc} s.t & C1:\sum_{j=1}^{N}r_{B,i,j} \end{array}\leq r_{B,i,\max},\\ \begin{array}{ccc} & & C2:r_{B,i,j},e^{\overset{-}{P_{B,i,j}}}>0, \end{array}\\ \begin{array}{ccc} & & C3:e^{\overset{-}{P_{B,i,j}}}\leq P_{\max}. \end{array} \end{array}$$

To solve the original problem (48), the maximum the lower bound $\sum_{j=1}^{N}\frac{r_{B,i,j}}{In2}[(\theta_{B,i,j}^{\left(1\right)}In\overset{-}{\gamma_{B,i,j}^{\left(1\right)}}$$+\;\beta_{B,i,j}^{\left(1\right)})+\left(\theta_{B,i,j}^{\left(2\right)}In\overset{-}{\gamma_{B,i,j}^{\left(2\right)}}+\beta_{B,i,j}^{\left(2\right)}\right)]$ is obtained by dual decomposition algorithm [46]. By using (51) and (52), we iteratively update the approximation parameters $\theta_{B,i,j}^{},\beta_{B,i,j}^{}$, then use the updated parameters for the next iteration until the results converge, as stated in the SCA approach above.

In each iteration, we solve the subproblem (53) by means of the Lagrangian dual method. The Lagrangian function is

(54) $$\begin{array}{c} L\left(r_{B,i,j},\overset{-}{P_{B,i,j}},\lambda ,\mu \right)=\sum_{j=1}^{N}\frac{r_{B,i,j}}{In2}\left[\left(\theta_{B,i,j}^{\left(1\right)}In\gamma_{B,i,j}^{\left(1\right)}+\beta_{B,i,j}^{\left(1\right)}\right)+\left(\theta_{B,i,j}^{\left(2\right)}In\gamma_{B,i,j}^{\left(2\right)}+\beta_{B,i,j}^{\left(2\right)}\right)\right]\\ +\lambda \left(r_{B,i,\max}-\sum_{j=1}^{N}r_{B,i,j}\right)+\mu \left(P_{\max}-e^{\overset{-}{P_{B,i,j}}}\right) \end{array}.$$

Based on the standard KKT condition [46], by deriving $r_{B,i,j}$ and $P\overset{-}{{}_{B,i,j}}$, we can obtain the optimal power allocation scheme of the subproblem in the form of λ and μ:

(55) $$P_{B,i,j}\left[t+1\right]=e^{\overset{-}{P_{B,i,j}\left[t+1\right]}}={\left[\frac{\theta_{B,i,j}^{\left(1\right)}+\theta_{B,i,j}^{\left(2\right)}}{\mu In2+\frac{\theta_{B,i,j}^{\left(1\right)}\rho_{B,i,j}^{\left(1\right)}H_{B,i,j}^{\left(1\right)}}{\rho_{B,i,j}^{\left(2\right)}H_{B,i,j}^{\left(2\right)}e^{\overset{-}{P_{B,i,j}\left[t\right]}}+I_{s}+r_{B,i,j}\left[t\right]N_{0}}}\right]}^{+},$$

(56) $$r_{B,i,j}\left[t+1\right]={\left[\frac{\lambda In2-\frac{\theta_{B,i,j}^{\left(1\right)}\rho_{B,i,j}^{\left(1\right)}H_{B,i,j}^{\left(1\right)}e^{\overset{-}{P_{B,i,j}}\left[t\right]}}{\rho_{B,i,j}^{\left(2\right)}H_{B,i,j}^{\left(2\right)}e^{\overset{-}{P_{B,i,j}\left[t\right]}}+I_{s}+r_{B,i,j}\left[t\right]N_{0}}-\frac{\theta_{B,i,j}^{\left(2\right)}\rho_{B,i,j}^{\left(2\right)}H_{B,i,j}^{\left(2\right)}e^{\overset{-}{P_{B,i,j}\left[t\right]}}}{I_{s}+r_{B,i,j}\left[t\right]N_{0}}}{N_{0}e^{\overset{-}{P_{B,i,j}\left[t\right]}}\left(\theta_{B,i,j}^{\left(1\right)}\rho_{B,i,j}^{\left(1\right)}H_{B,i,j}^{\left(1\right)}+\theta_{B,i,j}^{\left(2\right)}\rho_{B,i,j}^{\left(2\right)}H_{B,i,j}^{\left(2\right)}\right)}\right]}^{+}.$$

where ${\left[x\right]}^{+}=\max\left(0,x\right)$.

As $L(r_{B,i,j},\overset{-}{P_{B,i,j}},\lambda ,\mu )$ in (54) is not differentiable, the Lagrange multiplier λ and μ can be obtained by using the subgradient method iteratively as follows:

(57) $$\mu \left[t_{\delta }+1\right]={\left[\mu \left[t_{\delta }\right]-\delta_{\mu }\left(P_{\max}-P_{B,i,j}\right)\right]}^{+},$$

(58) $$\lambda \left[t_{\delta }+1\right]={\left[\lambda \left[t_{\delta }\right]-\delta_{\lambda }\left(r_{B,i,\max}-\sum_{j=1}^{K_{i}/2}r_{B,i,j}\right)\right]}^{+},$$

where $\delta_{\lambda }$ and $\delta_{\mu }$ are the step size of λ and μ in each iteration. The satellite beam channel allocation scheme is summarized as Algorithm 1. In the outer loop, we update the approximation parameters $\theta_{B,i,j}^{}$ and $\beta_{B,i,j}^{}$, and transform the original optimization problem into a solvable convex problem by logarithmic approximation. In the inner loop, we solve the transformed subproblem using the Lagrangian dual method. By updating the approximation parameters iteratively, we can finally obtain the optimal solution of original problem in (53).

### 5.4. Joint Power Allocation

In the above derivation, we obtain the power allocation schemes of the BSs and the satellite separately while fixing the parameters of another. Actually, the two networks will interfere each other as the entire bandwidth is reused in the whole system. Thus, the solution of one network will effect the solution of the other. To obtain the optimal solution of the whole system, an iterative algorithm is proposed as Algorithm 3. In each iteration, the Algorithms 1 and 2 will be performed based on the power allocation scheme $P_{B,i,j}\left[t\right]$ and $P_{S,l,J}\left[t\right]$ obtained in the last iteration. Then, the new obtained results will be used for the next iteration until the results converge. Note that in each iteration, the initialization steps in Algorithms 1 and 2 will be performed based on the results of last iteration, thus the computational complexity of Algorithm 3 is $O(L.{\left(K_{i}/2\right)}^{2}+I.N^{2})$.
**Algorithm 2** BS Channel Resource Allocation.1: **Initialize:** t=1,$\theta_{B,i,j}^{\left(1\right)}=\theta_{B,i,j}^{\left(2\right)}=1,\beta_{B,i,j}^{\left(1\right)}=\beta_{B,i,j}^{\left(2\right)}=0$; Initialize$P_{B,i,j}=0$; 2: **repeat** 3: Initialize$t_{\delta }=1$, λ>0,μ>0 4: Initialize $I_{s}$ 5:    **repeat** 6:       **for** J=1 to *N* do 7:          Update $P_{B,i,j}$ by using (55) 8:          Update $r_{B,i,j}$ by using (56) 9:       **end for** 10:    Update λ,μ by using (57) and (58) 11:    Set $t_{\delta }=t_{\delta }+1$ 12:    Until λ,μ converges 13: Set $P_{B,i,j}\left[t\right]=P_{B,i,j}\left[t+1\right]$ 14: Update$\theta_{B,i,j}^{\left(1\right)},\theta_{B,i,j}^{\left(2\right)},\beta_{B,i,j}^{\left(1\right)},\beta_{B,i,j}^{\left(2\right)}$ by using (51) and (52) 15: Set t=t+1 16: Until $P_{B,i,j}$,$r_{B,i,j}$ converges

**Algorithm 3** Joint Power Allocation.1: **Initialize:** Initialize t=1,$P_{S,l,J}\left[t\right]=0$,$P_{B,i,j}\left[t\right]=0$; 2: **repeat** 3:    **for** l=1 to *L* do 4:       Update$P_{S,l,J}\left[t+1\right]$ referring to Algorithm 1 5:    **end for** 6:    **for** i=1 to *I* do 7:       Update $P_{B,i,j}\left[t+1\right]$ referring to Algorithm 2 8:    **end for** 9:    Set $P_{S,l,J}\left[t\right]=P_{S,l,J}\left[t+1\right]$ 10:    Set $P_{B,i,j}\left[t\right]=P_{B,i,j}\left[t+1\right]$ 11:    Set t=t+1 12: Until $P_{S,l,J}$,$P_{B,i,j}$ converges 

## 6. Simulation and Analysis

To verify the effectiveness of the proposed DD-NOMA algorithm, MATLAB software is used to simulate the resource allocation of non orthogonal multiple access. Meanwhile, the throughput and outage probability performance are adopted as the performance metrics. In the experiments, the proposed DD-NOMA algorithm is compared with Channel Correlation coefficient-based NOMA (CC-NOMA) [29], cognitive Radio network-based OMA (CR-OMA) [47] and Dynamic Clustering-based NOMA (DC-NOMA) [19]. In specific, CC-NOMA selects two users with the largest channel correlation coefficient to pair and utilizes the alternating direction method of multipliers (ADMM) method to obtain the suboptimal power allocation solution. CR-OMA algorithm adopts orthogonal frequency division to access the satellite ground cognitive network and proposes a joint resource allocation optimization model based on efficiency and fairness. Under the constraint of maximum tolerable interference outage probability, the optimal resource allocation is solved by the closed form expression of power allocation and subchannel allocation. In DC-NOMA algorithm, a stagger and fold method is proposed for user pairing to avoid the situation that the difference of channel gain may be small. Then, the KKT optimization method is used to solve the optimal power allocation to maximize the total user capacity under the constraints of total transmission power and minimum user rate.

### 6.1. Simulation Parameter

In the simulation scenario, the access model of TSIN is composed of a LEO satellite and 50 base stations. The user’s position follows an independent homogeneous Poisson process. The BS communication channel adopts a Rayleigh fading model, and the satellite communication channel adopts an LES model. As shown in Table 2, the altitude of each BS is set at 10 m, and the altitude of the satellite is 1000 km. Define $d_{B}$ is the distance between the user and base station, then the path loss model expression is $52.87+37.6log10*d_{B}$. The path loss model of satellite channel adopts free space model, and the expression is $57.88+20log10*d_{S}$, where $d_{S}$ is the distance between the user and satellite.

### 6.2. Simulation Results and Analysis

**Figure 6:**
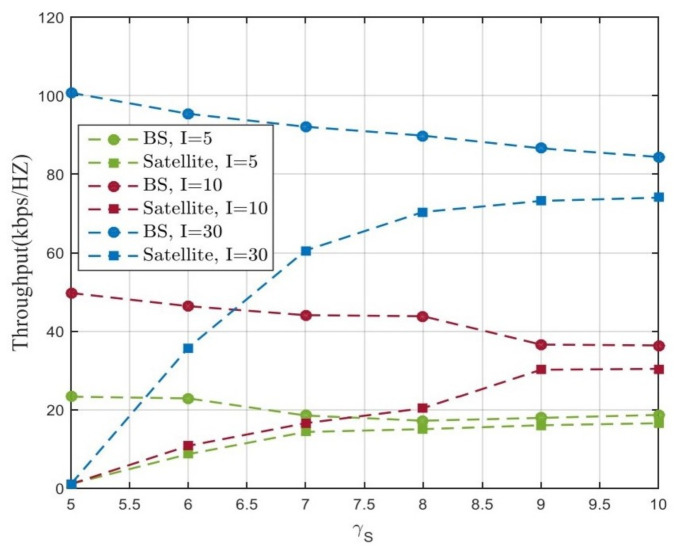
Comparison of BS capacity and satellite capacity with different $\gamma_{S}$.

In Figure 6, we investigate the variation of the system capacity with different $\gamma_{S}$ in three cases. We can observe that if less BS users are in the network, the decreasing speed of the capacity of BSs will be larger than the increasing speed of the capacity of the satellite at the first, and then the increasing speed will exceed the decreasing speed as the $\gamma_{S}$ continues to increase. If there are more BS users, either by increasing the number of BSs, as illustrated in Figure 6 where we increase *I* from 5 to 30, the decreasing speed of the BSs will always be less than the increasing speed of the satellite. Thus, with the help of the satellite, the network can provide service for more users simultaneously, especially for those users with bad BS channel conditions. Sacrificing part of the capacity of the users with good channel conditions, the system can provide better service for those users that have no access or bad access to the BSs by introducing the satellite to the system, and the total user number that can be served is also increased.

**Figure 7:**
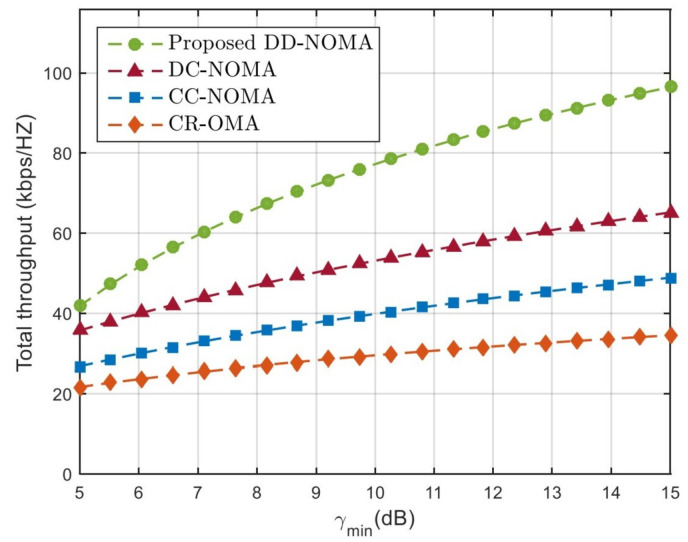
Total user throughput under different SINRs.

Figure 7 compares the total throughput performance of different algorithms, it can be seen that the increase of SINR threshold improves the total throughput of all algorithms, because the higher SINR threshold increases the power allocated by the user terminal, thus improving the total throughput. Compared with CC-NOMA and DC-NOMA, the proposed DD-NOMA has the best throughput performance. When the SINR threshold is 10dB, the total throughput of DD-NOMA is approximately 28% and 34% higher than that of CC-NOMA and DC-NOMA. The reason is that DD-NOMA couples users with large channel differences into a NOMA group, and uses the joint iterative algorithm to get the optimal resource allocation, better system capacity can be obtained. In contrast, CR-OMA can not achieve signal superposition on the same time-frequency resource, and its throughput performance is lower than other NOMA algorithms. In CC-NOMA, authors proposed a channel correlation coefficient algorithm to pair users with the largest difference in current channel conditions. However, the algorithm is local optimal, it can hardly guarantee the channel difference of remaining users. As a result, the throughput of remaining users would be reduced. Due to the lack of stable pairing guarantee mechanism in DC-NOMA algorithm, the total throughput is lower than the proposed DD-NOMA.

**Figure 8:**
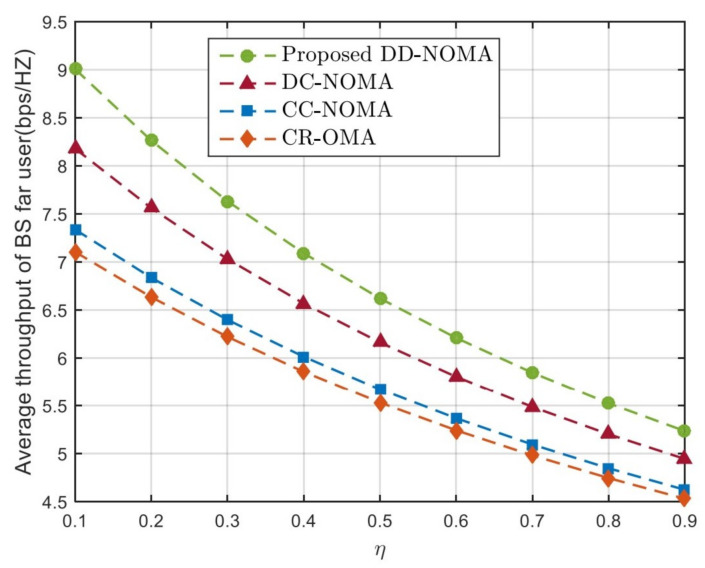
Average throughput of BS far users under different η.

**Figure 9:**
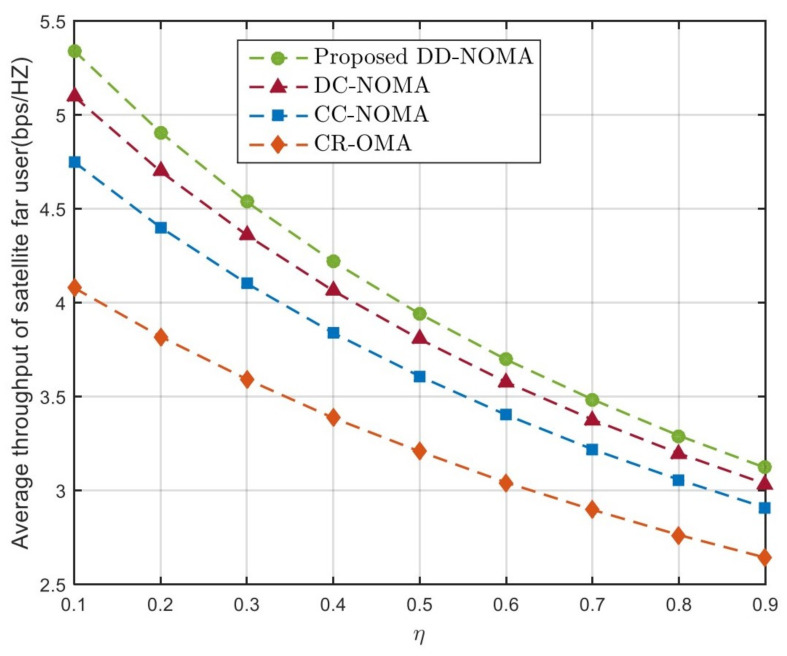
Average throughput of satellite far users under different η.

To verify the performance of the proposed DD-NOMA algorithm when the channel gain distribution is uneven, the definition of $\eta =\frac{d_{med}}{R}$, which can reflect the distribution uniformity of far and near users. Figure 8 and Figure 9 compare the average throughput of BS remote users and satellite remote users of the four algorithms under this parameter. It can be seen that the throughput of remote users decreases with the increase of η. The reason is that the larger the η, the more remote users are distributed at the cell edge, which reduces the channel gain of remote users and leads to the decrease of throughput. CR-OMA algorithm can not guarantee the throughput of remote users because it can provide resources for remote users while ensuring the demand of primary users. DC-NOMA uses the dislocation folding method to pair group users, which can ensure the diversity of users in the group, so its performance is better than CC-NOMA algorithm and CR-NOMA algorithm. The average throughput of the proposed DD-NOMA algorithm is 2.6%, 8.1% and 12.5% higher, respectively, than that of the DC-NOMA, CC-NOMA and CR-OMA algorithms when η=0.5. This is because the proposed user pairing algorithm can avoid users with similar channels from being divided into the same group when the channel gain distribution of users is unbalanced. Meanwhile, the optimal power control factor is given to guarantee the throughput of edge users, which can effectively improve the throughput of far users.

**Figure 10:**
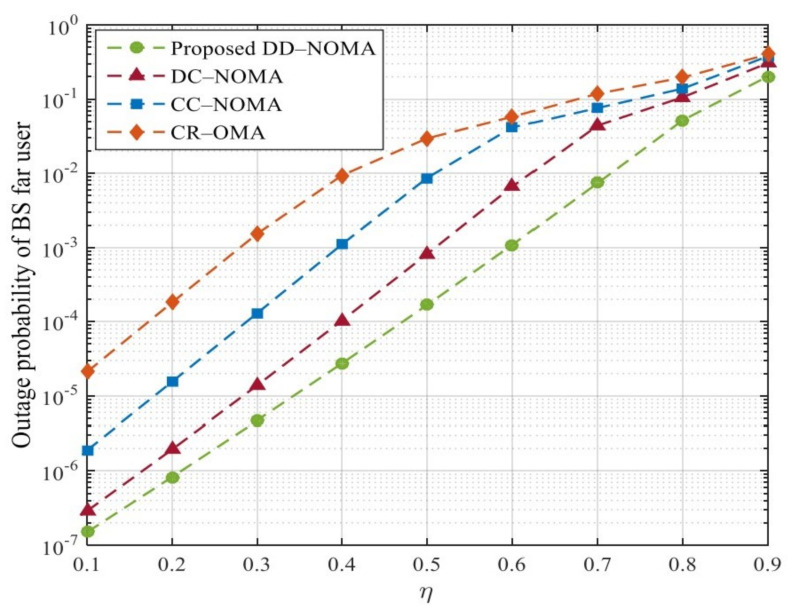
Outage probability of BS far users under different η.

**Figure 11:**
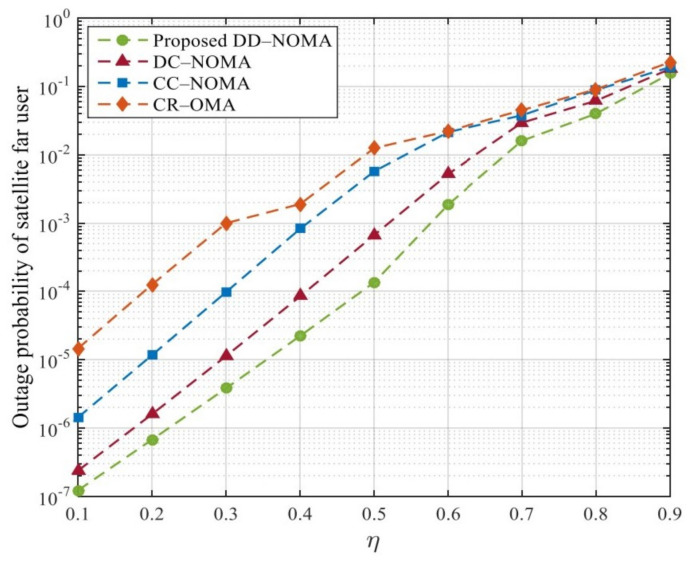
Outage probability of satellite far users under different η.

Figure 10 and Figure 11 compare the outage probabilities of the four algorithms; it can be seen that the outage probabilities of the BS far user and the satellite far user increase with the increase of the distance between the far user and the BS or the beam center. This is because with the increase of the distance, the more serious the channel fading of the far user, the lower the transmission rate, and the higher the outage probability. DD-NOMA algorithm has the best outage probability performance. When η=0.5, the outage probability of satellite far users is only 0.01%. The reason is that in order to meet the outage performance requirements of users with poor link quality, the optimal power control factor designed can ensure the minimum transmission rate of far users and reduce the outage probability. The far user in CR-OMA systems only achieves a diversity gain of primary user, which is worse than DC-NOMA and CC-NOMA. This is because in CR-OMA systems, only after primary user’s QoS requirement is strictly satisfied, far user is allowed to be admitted into the system. Note that increasing η from 0.4 to 0.5 can increase the outage probability. This is because when η=0.5 , the path loss of far users become larger, the transmission rate is reduced, the CR method designed cannot ensure the minimum transmission rate of far users, which in turn affects the outage probability. In contrast, DC-NOMA can ensure that both users’ outage probability are lower than that in CR -OMA. In addition, it can be seen that when the η increases, the diversity gain of far user is reduced in DC-NOMA and CC-NOMA. The main reason is that when the η is high, the transmission rate is reduced, thus the far user may fail to detect near user message, which leads to the implementation failure of SIC.

## 7. Conclusions

To improve the fairness and resource utilization of user access, a NOMA method based on dynamic divide grouping is proposed. By constructing an optimization model of joint user grouping, power control and resource allocation, the interference problem caused by the integration of satellite network and terrestrial network is solved. Aiming at the time-varying channel gain of satellite-terrestrial link, the relationship model between user elevation angle, beam angle and distance is established, and the dynamic user pairing algorithm is proposed to ensure the effective pairing of users. In order to obtain the optimal resource allocation, the power control factor is derived according to the instantaneous channel gain of users. To solve the non-convex problem, the original problem is transformed into a convex problem by variable substitution, and the optimal solution is obtained by dual algorithm. Simulation results show that the proposed method can effectively improve the system throughput and outage probability performance.

## Figures and Tables

**Table 1:** NOMA for integrated terrestrial–satellite networks.

Reference	System Model	Usering Pairing	Optimization Method	Limitations
[15,16]	Multi BS cell + K users	Mean clustering	Branch-and-bound method	Fixed power allocation lead to suboptimal solution
[18]	Multi BS cell + K users	Greedy algorithms	Pareto-boundary commuted using reformulation	Fixed power allocation lead to suboptimal solution
[20]	Multi BS cell + K vehicles	Random	Analytic techniques	Channel difference of user pairing cannot be guaranteed
[21]	Multi BS cell + K users	Random	Successive convex approximation	Random user pairing will affect the transmission rate of users
[24]	Cognitive radio networks	Random	Analytic techniques	No user pairing method and CR introduces signal interference between networks
[26]	A fixed satellite + BS + k users	No	Slotted Aloha method	Users number is limited by OMA method
[27]	Satellite + k UAVs	Random	Heuristic algorithm	Random user pairing will affect the transmission rate of UAV
[28]	Satellite + k users	Random	Iterative optimization algorithm	Channel difference of user pairing cannot be guaranteed
[29]	Satellite + k users	Channel correlation coefficient	A suboptimal algorithm based on alternate direction	User pairing method will not guarantee stable pairing relationship
[30]	Fixed satellite + k earth stations	Stagger and fold method	Taylor expansion and penalty function methods	The impact of satellite time-varying links on user pairing isnot analyzed
[34]	Satellite + BS cell + K users	Channel correlation coefficient	Iterative scheme based on Karush-Kuhn-Tucker approaches	User pairing method will not guarantee stable pairing relationship

**Table 2:** Simulation parameter.

Parameters	Values
Orbital altitude	1000 km
Number of beams	5
BS number of beam coverage	10
Number of BS subchannels	10
Number of beam subchannels	50
User terminal height	1.5 m
BS height	10 m
Coverage radius of BS	5 km
Coverage radius of beam	50 km
The maximum power of group $P_{\max}$	18 dBm
Satellite antenna gain	25 dBi
BS antenna gain	17 dBi
User antenna gain	0 dBi
Noise power spectral density	−174 dBm/HZ
